# Impact of single room design on the spread of multi-drug resistant bacteria in an intensive care unit

**DOI:** 10.1186/s13756-017-0275-z

**Published:** 2017-11-15

**Authors:** Teysir Halaby, Nashwan al Naiemi, Bert Beishuizen, Roel Verkooijen, José A. Ferreira, Rob Klont, Christina vandenbroucke-Grauls

**Affiliations:** 1 Laboratory for Medical Microbiology and Public Health, Boerhaavelaan 59, 7555 BB Hengelo, The Netherlands; 2 Department of Medical Microbiology & Infection Control, VU University Medical Center, Amsterdam, The Netherlands; 3 Medical Microbiology and Infection Control, Ziekenhuisgroep Twente, Almelo, The Netherlands; 4 Department of intensive care, Medisch Spectrum Twente, Enschede, The Netherlands; 5 Department of Statistics, Informatics and Modelling, National Institute for Public Health and the Environment, RIVM, Bilthoven, The Netherlands

## Abstract

**Background:**

Cross-transmission of nosocomial pathogens occurs frequently in intensive care units (ICU). The aim of this study was to investigate whether the introduction of a single room policy resulted in a decrease in transmission of multidrug-resistant (MDR) bacteria in an ICU.

**Methods:**

We performed a retrospective study covering two periods: between January 2002 and April 2009 (old-ICU) and between May 2009 and March 2013 (new-ICU, single-room). These periods were compared with respect to the occurrence of representative MDR Gram-negative bacteria. Routine microbiological screening, was performed on all patients on admission to the ICU and then twice a week. Multi-drug resistance was defined according to a national guideline. The first isolates per patient that met the MDR-criteria, detected during the ICU admission were included in the analysis. To investigate the clonality, isolates were genotyped by DiversiLab (*bioMérieux*, France) or Amplified Fragment Length Polymorphism (AFLP). To guarantee the comparability of the two periods, the ‘before’ and ‘after’ periods were chosen such that they were approximately identical with respect to the following factors: number of admissions, number of beds, bed occupancy rate, per year and month.

**Results:**

Despite infection prevention efforts, high prevalence of MRD bacteria continue to occur in the original facility. A marked and sustained decrease in the prevalence of MDR-GN bacteria was observed after the migration to the new ICU, while there appear to be no significant changes in the other variables including bed occupancy and numbers of patient admissions.

**Conclusion:**

Single room ICU design contributes significantly to the reduction of cross transmission of MRD-bacteria.

## Background

Cross-transmission of nosocomial pathogens has been shown to occur frequently in intensive care units (ICU) [1]. It may be promoted by several factors including environmental source [2], invasive procedures, and understaffing [3]. Bacterial cross-transmissions account for a significant part of ICU-acquired infections [4], the majority of which are associated with Gram-negative (GN) microorganisms [5]. GN-infections in turn lead to substantial morbidity, mortality and costs [6]. Interventions aimed at reducing the spread of nosocomial pathogens include contact precautions and isolation of patients, especially when multi-drug resistant (MDR) organisms are involved [7] and hand hygiene [8], which has been considered the most important control measures [9]. Despite evidence that transmission of pathogens by way of health care workers’ hands is a major cause of nosocomial infections [10], compliance with policies and procedures for infection control has been uniformly poor [11]. Nursing patients in single-patient rooms can improve hand washing compliance and facilitate cleaning and decontamination and thereby contribute to infection control [12].

In this retrospective study we describe the long-term persistence and transmission of MDR-GN organisms in an ICU despite extensive infection control precautions. We present evidence for the role of the single room design of the new facility to which the ward was eventually moved in their control.

## Methods

### Setting

**Fig. 1:**
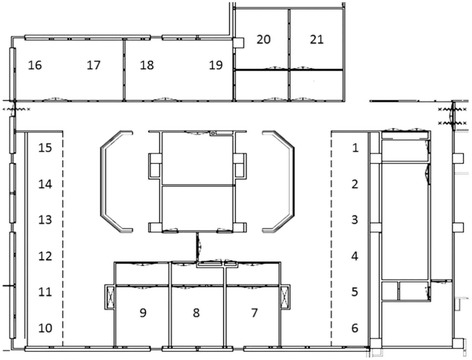
Floor plan of the ICU before conversion: 1–6 and 10–15: beds situated in the open bay; 7–9 and 20–21: single rooms with controlled ventilation and with anteroom; 16/17 and 18/19: rooms without controlled ventilation an without anteroom

The study covered two periods: a first period between January 2002 and April 2009 (old-ICU) and a second between May 2009 and March 2013 (new-ICU, single-room). In the first period patients were nursed in an ICU with 21 beds: five in single ventilated rooms and with ante-room, four in two double rooms without ante-rooms, and 12 in an open bay (Fig. 1). The total number of beds in use was 18, since a maximum of nine out of the 12 open bay beds was used for admissions at any given time.

**Fig. 2:**
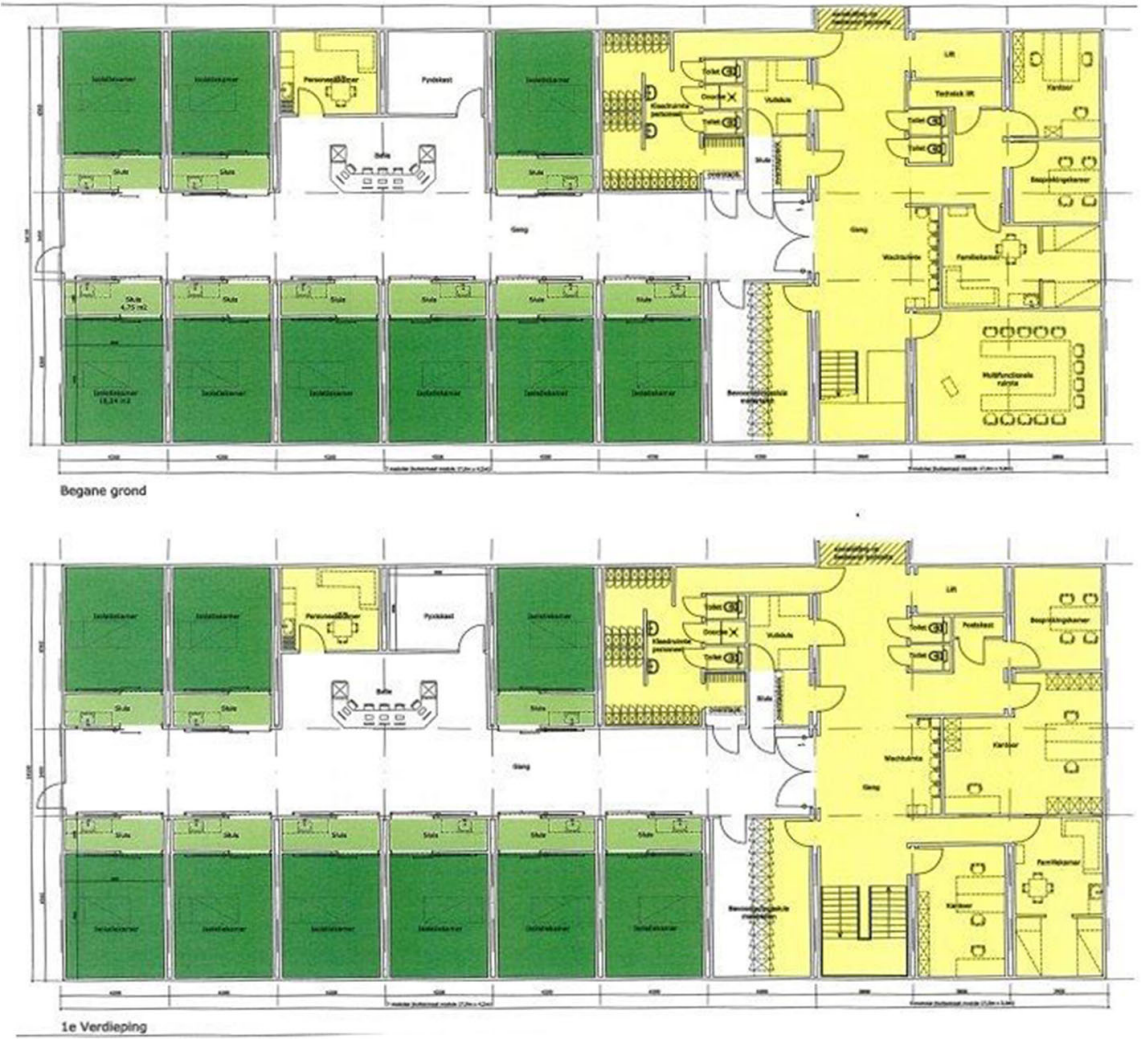
Plan of the new ICU consisting of two identical floors with 9 patient rooms each. Single patient rooms with anteroom are indicated by dark green and light green, respectively

This ICU was closed on two occasions: from January through May 2003 because of an ongoing outbreak with ESBL-producing *Klebsiella pnumoniae* (ESBL-Kp) which started in 2001 [13], and between January and March 2008 because of an outbreak with multi-drug resistant *Acinetobacter baumannii* (MDR-Ab). Because of these outbreaks and because there was evidence of persistent colonization and spread of other MDR-GN among patients in the ICU despite extensive infection control efforts, it was ultimately decided to transfer the ICU in May 2009 to a newly built ICU, which consisted of a two-floor unit, each with 9 single rooms with controlled ventilation and ante-room (Fig. 2). The two floors were identical regarding treatment facilities and casemix. Initially only 16 beds were used. In January 2011 the number beds in use was increased to 18. Only new patients were admitted to the new ICU and no patients we transferred from the old ICU during migration.

### Surveillance

Routine microbiological screening started in February 2002 and continued through the whole study period. Screening was performed on all patients on admission to the ICU and then twice a week. Screening was done by culture of throat and rectal-swab specimens and, in intubated patients, of tracheal fluid samples. When clinically indicated, samples were also obtained from relevant body sites, such as wounds. MDR-GN strains were stored at −70 °C.

### Bacteriological methods

From January 2002 until April 2010, species identification was routinely performed by classical biochemical methods and the API 20E system. Antimicrobial susceptibility testing was performed by the agar dilution method according to the National Committee on Clinical Laboratory Standards (NCCLS), now called Clinical and Laboratory Standards Institute (CLSI) [14]. From April 2010 on, the Vitek 2 Advanced Expert System (bioMérieux, France) was used to identify strains, to determine antimicrobial susceptibility using the EUCAST breakpoints [15], and to perform phenotypic screening for ESBL. ESBL confirmation was performed by the double disk synergy test with cefotaxime and/or ceftazidime, and clavulanic acid [16].

### Infection control

Before the outbreak, infection control measures in the ICU were implemented according to a national guideline [17]. Infection prevention measures were mainly based on the so-called “work island” principle, which means contact precautions in the area surrounding the ventilated patient bed, including cleaning and disinfection and hand hygiene before entering and by leaving the patient area.

**Fig. 3:**
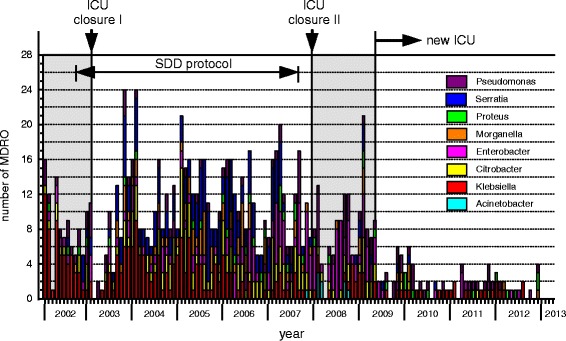
Occurrence of MDR-resistant bacteria (each counted once per patient) between January 2002 and March 2013 with indication of the main infection control measures that were taken on the ICU. ICU closure I: during January–May 2003 for thorough cleaning and disinfection; ICU closure II: temporary closure for new admissions because of an outbreak with multi-drug resistant *A. baumannii*. After all beds became available through discharges the unit including equipment was decontaminated with vaporized hydrogen peroxide; new ICU opened with single-bed rooms

When an increase in the number of patients colonized with ESBL-Kp was noticed in August 2001, an outbreak management team was formed, including an infection control nurse, an ICU medical officer, a consultant microbiologist, and an ICU nurse. Infection control practices were reinforced, including labelling and isolation of ESBL-Kp-positive patients in the single-patient rooms, cohort nursing of ESBL-Kp-colonised patients to the two-bedded rooms when more patients were found colonized, and disinfection of hospital equipment and high-touch surfaces. Since the outbreak remained uncontrolled despite these measures (Fig. 3), an intensified infection control programme was started. This included from September 2002, the use of a ‘short stay’ four-bedded unit outside the ICU area for patients expected to be admitted to the ICU for fewer than 72 h. Secondly, from October 2002 onwards, patients admitted to the ICU received selective decontamination of the digestive tract (SDD). The aim of the SDD treatment in this setting was to reduce colonization of the digestive tract with resistant bacteria [18]. SDD was given as topical mixture of nonabsorbable antibiotics including tobramycin, colistin and Amphotericin B (respective doses: 80, 100, and 500 mg), applied on the buccal mucosa and as a suspension administered via a nasogastric tube in the gastrointestinal tract, four times a day [19].

Finally, the ICU was temporarily closed from January through May 2003 for thorough cleaning and disinfection, during which period patients were admitted to a temporary, 16-bed ICU. The same infection control policy from the closed ICU was continued. No new patients with ESBL-Kp were detected during this period, until one week before moving the ICU back to the main location. After the ICU was moved back to the main location, an increase in the incidence of ESBL-Kp positive patients was noted (Fig. 3). In 2005 a decrease in the incidence was observed; however, the outbreak remained uncontrolled. In 2007, it was concluded that radical facility changes in design and infection control policy were needed for optimal infection control practices. Short-term changes that followed within the following year included SDD discontinuation (April 2007), the appointment of additional infection control practitioners, and the promotion of a high level of compliance with infection control measures.

Between January and March 2008 the ward was closed due to an outbreak with MDR-Ab. Rigorous infection control measures were implemented including the grouping of MDR-Ab-positive patients in single rooms with controlled ventilation, education of staff, enforcement of hand hygiene and surface decontamination. In addition, the ward was temporarily closed for new admissions. After all beds became available through discharges, the unit including equipment was decontaminated with vaporized hydrogen peroxide (VHP), according to manufacturer’s instructions (Infection Control BV, Eemnes, the Netherlands). Short-term changes were implemented, including the reduction of the number of beds to 16, and the unit was re-opened on April the 3rd 2008. In April 2009 the ICU was moved to a semi-permanent (http://www.cadolto.com/en/products/healthcare_buildings/hospitals) single-room unit (Fig. 2). Nurse-to-patient ratio (0,66) did not change. The same infection control protocols were maintained. In the single-room unit, a hand washing sink was located in each ante-room and an alcohol-based hand rub dispenser in each ante-room and at the bedside*.*

### Retrospective microbiological analysis

In 2014, a retrospective study was undertaken on existing laboratory databases to collect data on the occurrence of MDR-GN, including ESBL-Kp, *Citrobacter* spp., *Proteus* spp., *Enterobacter* spp., *Serratia* spp., *Morganella* spp., *Pseudomonas* spp. and *Acinetobacter* spp. Multi-drug resistance among Gram-negative bacteria was defined according to a national guideline (Table 1) [20].

**Table 1:** Definition of MDR Gram-negative bacteria [20]

	ESBL	carbapenems	fluoroquinolons	aminoglycosides	ceftazidime	piperacillin	cotrimoxazole
*K. pneumonia*	A	A	B	B			
other Enterobacteriaceae^a^			B	B			B
*P. aeruginosa*		C	C	C	C	C	
*A. baumannii*		A	B	B	B		

*MDR* multi-drug resistant

^a^ Proteus, Morganella, Serratia, Citrobacter, Enterobacter spp.

A: presence of ESBL production or resistance against this antibacterial agent or group is sufficient to define the microorganism as being MDR

B: resistance against 2 antibacterial agents or against at least 2 of the indicated groups is required to define the microorganism as being MDR

C: resistance against 3 antibacterial agents or against antimicrobial agents from at least 3 of the indicated groups is required to define the microorganism as being MDR

The first isolates per patient, that met the MDR-criteria, detected during the ICU admission were included in the analysis.

Since the dilutions values of antimicrobial agents that were tested in the agar dilution method were available in the Laboratory Information System (LIS), it was possible to compensate for the change from CLSI to EUCAST by retrospectively redefining the breakpoints of the tested isolates to meet the MDR-criteria. During the whole study period, ESBL identification was performed on *K. pneumonia*, *K. oxytoca* and bacteriemic *P. mirabilis* isolates that screened suspected [14]. Bacteriemic *P. mirabilis* isolates were not detected [21], hence, no ESBL confirmation was performed. So, from the ESBL-positive Enterobacteriaceae only *K. pneumonia* isolates were included in the analysis. The other Enterobacteriaceae were included not as whether or not carrying ESBL but when meeting the HRMO criteria.

**Fig. 4:**
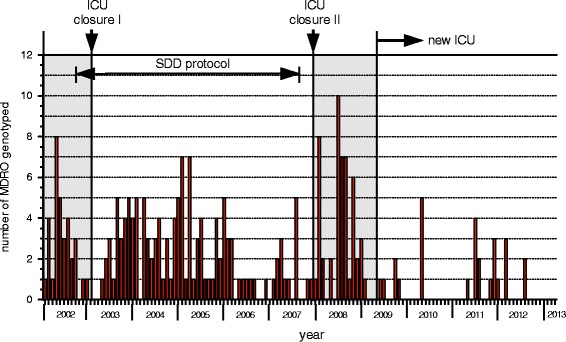
Frequency distribution of the MDR Gram negative bacteria that were used for genotyping

Clonality of stored ESBL-Kp isolates obtained between 2002 and 2007 was retrospectively investigated by DiversiLab (bioMérieux, France) [21]. Available data from typing of *Enterobacter*, *Acinetobacter*, *P. aeruginosa*, *Citorbacter freundii*, *E. coli* and ESBL-Kp isolates obtained after 2007, prospectively investigated by DiversiLab or AFLP [22], were also included in this study. Frequency distribution of the MDR Gram-negative bacteria that were used for genotyping is shown in Fig. 4.

### Statistical analysis

Our objective was to investigate whether the introduction of a single room policy resulted in a decrease in the number of transmissions of MDR within the ICU. For this purpose we compared a period before the introduction of the policy and a period following it with respect to the occurrence of representative MDR isolates of the species *Citrobacter*, *Enterobacter*, *Morganella*, *Proteus*, *Serratia* and *Pseudomonas.* To guarantee the comparability of the two periods, the ‘before’ and ‘after’ periods were chosen such that they were approximately identical with respect to the following factors: number of admissions, number of beds, bed occupancy rate, per year and month (the average length of stay per month can be dispensed with since it is determined by the average number of admissions and the occupancy rate). A data set derived from the NICE database (National Intensive Care Evaluation, https://www.stichting-nice.nl) was combined with the laboratory data and upon examination of the monthly figures related to numbers of admissions, number of beds and bed occupancy it was decided to compare the periods from April 2008 to April 2009 and from May 2009 to December 2010, during the whole length of which the number of beds was kept at 16, as the ‘before’ and ‘after’ periods for the main part of the analysis. A third period (a second ‘after’ period) from January 2011 to March 2012, in which the capacity of the ICU was increased to 18 beds, was used for subsidiary analyses.

The two periods were compared with respect to a single variable (e.g. number of transmissions of a given bacterium or bed occupancy) by a permutation test with month as a ‘block factor’ based on a so-called *sum statistic* [23]. This is the sum, over the available months, of the monthly differences in the average values of the variable in the two periods. The blocking by month should correct for eventual seasonal patterns in the number of transmissions. In order to compare the two periods with respect to the transmissions of the six different species *simultaneously* we used a generalization of the permutation test based on the sum of the six sum statistics corresponding to the six bacteria. The rationale for using this test was the “principle of coherence” [24]: if the intervention does have a positive (or at least non-negative) effect then that effect consists of a decrease (or at least non-increase) in the number of all the bacteria. All the tests were two-sided. Despite several tests being carried out, no multiple testing corrections are presented because, by the nature of the data and of the hypotheses tested, the *p*-values are either unequivocally large or unequivocally small. Statistical analyses were carried out with programs written in R [25], which may be obtained from the authors upon request.

## Results

The numbers of patients carrying MDR-*Citrobacter* spp., *Proteus* spp., *Enterobacter* spp., *Serratia* spp., *Morganella* spp. and *Pseudomonas* spp. and ESBL-Kp are shown in Fig. 3.

### ESBL-Producing *K. pneumoniae*

Between January 2002 and March 2013, 225 patients with ESBL-Kp were identified (Fig. 3). Typing of 163 isolates (one isolate per patient) by REP-PCR revealed that the majority was clonally related. Of these isolates 121, obtained between January 2002 and July 2007, were found to be identical [21], while 42 appeared unrelated to the major clone. Typing of 10 out of the 17 ESBL-Kp isolates obtained between March 2008 and April 2009 (after disinfection of the old ICU with VHP and before the move to the new ICU), showed no clonal relation of these isolates to the outbreak strain. However, two clusters of strains were identified: one of two strains (isolated on 3/11/2008 and 11/12/2008), and the other of three strains (isolated on 18/12/2008, 29/12/2008 and 3/1/2009). The remaining five isolates had different patterns.

### A. Baumannii

In October 2007, a patient known to harbour MDR-Ab was transferred from a Turkish hospital to the ICU and was directly placed in a separate room in strict isolation. During his admission, which lasted three weeks, and after discharge, no spread of the MDR-Ab was seen, until early in January 2008, when two patients were found to carry a strain of MDR-Ab. The patients were placed in separate rooms with controlled ventilation in strict isolation. During the following two weeks, MDR-Ab was detected in three more patients. Genotyping showed clonal relationship between strains from the five patients, one strain from the index patient and seven from the ICU environment. The unit was closed in the third week of January and re-opened in March 2008.

### Other MDR-gram negative organisms

Between March 2008 and April 2009, 46 patients carrying MDR *E. cloacae* were identified. Typing of 23 isolates obtained between April and August 2008, with Diversilab [26] revealed two clusters: one of 7 and one of 13 strains. The remaining three strains had different patterns.

**Fig. 5:**
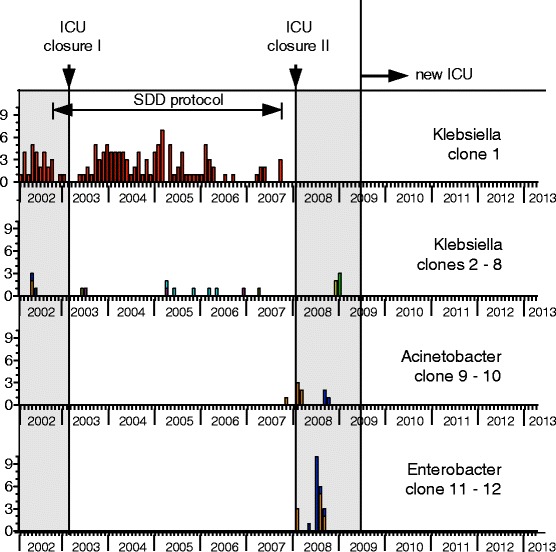
Data from typing of *Enterobacter*, *Acinetobacter* and ESBL-Kp isolates. Cross transmission of new microorganisms re-emerged and persisted after the old ICU was reopened in March 2008. The second closure ended the cross transmission of ESBL-Kp and MDR-Ab

After the old ICU was reopened in March 2008, cross transmission of new microorganisms re-emerged and persisted as evidenced by genotyping of ESBL-Kp (unrelated to the major clone) and MDR *E. cloacae* (Fig. 5).

### New ICU period

**Fig. 6:**
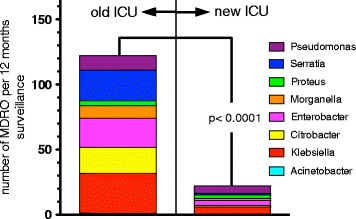
Plot diagram showing a decrease in the yearly numbers of MDR organisms isolated after the move to the new ICU. Data sets before and after the move consist of 88 and 47 months surveillance, respectively

**Fig. 7:**
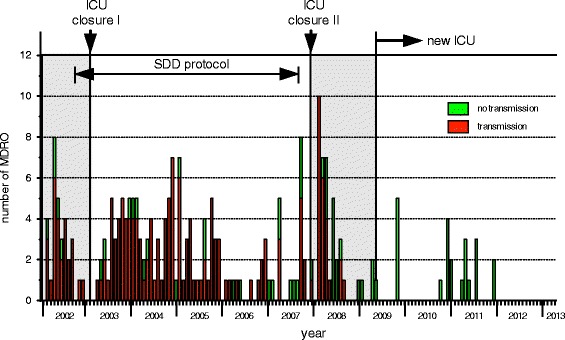
Pooled data from typing of *Enterobacter*, *Acinetobacter*, *P. aeruginosa*, *C. freundii*, *E. coli* and ESBL-Kp isolates. In the new ICU no transmission was observed

After the migration to the new ICU, a marked decrease in the prevalence of MDR-GN was observed (Fig. 6). Available data from typing of *Enterobacter*, *Acinetobacter*, *P. aeruginosa*, *C. freundii*, *E. coli* and ESBL-Kp isolates obtained after the migration showed no transmission (Fig. 7).

The test comparing the ‘before’ (April 2008 to April 2009) and ‘after’ (May 2009 to December 2010) periods regarding the numbers of transmissions of the six bacteria jointly yielded a *p*-value of 0.001. With respect to the number of admissions, the comparison between the ‘before’ and ‘after’ periods and between the ‘after’ period and *the second* ‘after’ period (January 2011 to March 2012) no significant changes were observed (*p*-values of 0.17, and 0.34).

With respect to bed occupancy, testing for differences between the ‘before’ and ‘after’ periods yields a p-value of 0.99. In contrast, there was evidence for a difference between the ‘after’ period and *the second* ‘after’ period (increase in the capacity of the ICU from 16 to 18 beds), with a p-value of 0.007.

Comparing the ‘before’ and ‘after’ periods with regard to each species one at a time, p-values of 0.0015, 0.0005, 0.37, 0.99, 0.25, and 0.39 for *Citrobacter*, *Enterobacter*, *Morganella*, *Proteus*, *Serratia* and *Pseudomonas*, respectively, were found.

## Discussion

Our results provided strong evidence that the single room policy as an infection control strategy has contributed significantly to the control of cross transmission of resistant pathogens in the ICU.

In this study we analysed the history of an ICU which was affected by a protracted clustered occurrence of MDR bacteria despite extensive infection control precautions. Control was ultimately achieved by closing the ward and moving it into a new single room designed facility.

Despite combined interventions, including education to improve adherence to hand hygiene practices, use of contact precautions, isolation of ESBL-Kp positive patients and temporary ward closure in early 2003, the colonization by endemic ESBL-Kp, as evidenced by genotyping with DiversiLab, and by other MDR-Gram negative bacteria was observed soon after the unit was re-opened. Educational meetings were held and hand hygiene was emphasized on several occasions. Recorded observations about hand hygiene performance, and adherence to hygiene protocols were not part of the experimental design, hence could not be described during the study period. Even if lack of adherence to hand washing protocols alone may not explain the failure to halt transmission [27], breaches in hand hygiene may have promoted it [28].

Although the importance of colonization pressure in transmission of MDR-GN bacteria has not fully been estimated [29], our data suggest that the increased number of colonized patients has contributed to the persistence of MDR-bacteria. Not only was the proportion of colonized patients high, patients were also colonized with multiple MDR-GN bacteria. Selection of these bacteria may have been facilitated by the start and the prolonged use of SDD. Prior to the introduction of SDD, most ESBL-Kp isolates were resistant to tobramycin, and upon exposure to colistin, heteroresistant subpopulations may have been selected for. In addition, the proportion of tobramycin resistance among pathogens intrinsically resistant to colistin (*Proteus*, *Morganella*, and *Serratia* spp.) increased under the use of SDD, and decreased after stopping SDD [21]. Abundant carriage of these MDR-bacteria under SDD, i.e. colonization pressure [30] may have enhanced the risk of their spread and acquisition.

Although temporary ward closure has been shown to control ESBL-Kp outbreaks adequately [31], in our case the ESBL-Kp outbreak persisted after closure of the ICU early in 2003. The reason for this is not clear; no common environmental source was identified, and cultures obtained from the hands of nursing and medical staff performed on one occasion were negative. After the second ward closure and decontamination with HPV early in 2008, clonal spread of the ESBL-Kp or MDR-Ab was not observed again. However, transmission and persistence of new MDR-GN bacteria after the ward was reopened continued to occur, as evidenced by typing of ESBL-Kp and *E. cloacae* strains.

Single-bed room design has been shown beneficial in reducing contact transmission and acquisition of resistant bacteria in several studies [32–36]. It enables the separation of patients upon admission and prevents transmission from unrecognized carriers of pathogens. By design, single rooms are furnished with a conveniently located sink in each, provided by sufficient and accessible alcohol-based hand-rub dispensers. Affecting staff behavior by single-room design has in one study been found a possible element that contributed to higher hand hygiene compliance, compared to an open plan ICU [32]. Another study showing a substantial reduction in transmission of some microorganisms after converting the ICU to private rooms, has attributed the observed reduction to better hand hygiene by hospital staff, rather than to the move to a new and uncontaminated enviromnemt [33]. The results from our study supports these findings. Although adherence to hand hygiene practice was not measured, ending of cross-transmission occurred only after the move to the new ICU (Fig. 6).

In this study, after the move to the single room unit, a clear and sustained decrease in the prevalence of the MDR-GN bacteria was observed, except for MDR-*P. aeruginosa* which, however, could not be explained by cross transmission since the genotyping of five isolates between July and October 2009 revealed no similarities. Statistical analysis testing for differences between the periods before and after the move to the single room unit showed good evidence that the single room policy was very effective in controlling the cross transmission of the MDR bacteria in this ICU.

## Conclusion

Protracted clustered occurrence of MDR bacteria in an ICU despite extensive infection control precautions, including temporary ward closure on two occasions, was ended only by the transformation of the unit into a single-room unit. Single room ICU design significantly contributed to the reduction of cross transmission of MRD-bacteria.
